# Supplementary material for: Fractional Moore-Gibson-Thompson heat transfer model with nonlocal and nonsingular kernels of a rotating viscoelastic annular cylinder with changeable thermal properties
Source: PLoS One. 2022 Jun 21;17(6):e0269862. doi: 10.1371/journal.pone.0269862 (PMC9212188; doi:10.1371/journal.pone.0269862)
Supplement: Attachment — Submitted filename: Response to Reviewer.docx [file pone.0269862.s001.docx]

**Response to Reviewer(s)' Comments**

**Article ID**: PONE-D-21-36348

Dear Prof.

Mohammad Mehdi Rashidi
Academic Editor
PLOS ONE

Thank you for giving me the opportunity to submit a revised draft of my manuscript titled " Fractional Moore-Gibson-Thompson heat transfer model with nonlocal and nonsingular kernels of a rotating viscoelastic annular cylinder with changeable thermal properties " to PLOS ONE.

We appreciate the time and effort that you and the reviewers have dedicated to providing your valuable feedback on my manuscript. We are grateful to the reviewers for their insightful comments on my paper. We have been able to incorporate changes to reflect most of the suggestions provided by the reviewers. We have highlighted the changes within the manuscript.

Here is a point-by-point response to the reviewers’ comments and concerns.

**Comments from Reviewer 1**

Thank you for your comments. We have studied your comments carefully and made major correction, which we hope meet with your approval. We answer your questions or comments in details in the following texts.

Reviewer #1: In manuscript No. PONE-D-21-36348, a thermoelastic fractional heat conduction model was developed based on the Moore-Gibson-Thompson equation to examine the axial symmetry problem of a viscoelastic orthotropic hollow cylinder. Some new results are reported. However, the following comment should be considered,

1- Please enhance the abstract with some quantitative results. For instance, it is mentioned that ‘The results showed that the effective variables have a significant effect on the responses to the variables of the studied fields.’, please mention what is a significant effect?

Reply: Thanks to the reviewer for the good suggestion. The summary is enhanced by some quantitative results.

The effect of various parameters, such as the fractal order, for example, on the responses of the studied field variables is explained in detail in the discussion section and summarized in the conclusions section.

2- - Also, please provide important quantitative results in the conclusion.

Reply: The conclusion is improved as the most important quantitative results obtained from the discussion are presented.

3- - Please provide a ‘Nomenclature’.

Reply: Thanks for the good suggestion. The nomenclature is presented at the beginning of the manuscript

4- - It seems that some figures can be combined. For example, the authors can join Figs. 13 to 16 to reduce the number of figures.

Reply: Thanks for the suggestion.

But we want to point out that viscosity is an important part of the contributions of the present work in this manuscript in addition to other contributions that have been made such as fractional differentiation. For this reason, the study of this case cannot be excluded, the Figures associated with it cannot be combined and cannot be dispensed with. We agree with the references' point of view that there are many Figures in the paper.

5- - The authors should provide the details of validation in Section 8.

Reply: Thanks for the good suggestion. Verification details are provided in the discussion section 8.

6- The literature review can be updated with some other studies on fluid flow and heat transfer inside tubes and channels. For instance, <https://doi.org/10.1016/j.cep.2015.08.009> and <https://doi.org/10.1016/j.applthermaleng.2016.07.173>

Response: The presentation of the paper, especially the introduction and discussion sections, was improved, and the references mentioned were used in addition to other recent papers.

7- Finally, I would suggest the authors double-check all sentences for English or ask someone to help with the writing.

Response: We thank the reviewer for his notes. The entire manuscript was revised, from the abstract and introduction to conclusions and references. Many parts of it have been reworked.

**Comments from Reviewer 2**

Thank you for taking the time to provide your valuable suggestions and review the manuscript. We have carefully studied your suggestions and made important changes. We have answered your questions and helpful comments in depth. We believe that you will agree with them on the following points:

Reviewer #2: Review Report:
The present manuscript is very interesting, but it needs some minor revisions before its publication as follows:

1. The literature review about the Atangana-Baleanu fractional derivative is not complete. Please review recently published papers.

Reply: Thanks for the good suggestion. The literature on the Atangana-Baleanu partial derivative has been reviewed and updated. Recently published papers have been added to the Introduction section.

2. How do the authors validate their results?

Reply: Thanks for the good suggestion. Verification details are provided in the discussion section 8.

3. Please check the comma and the point at the end of each expression. Please see Eq. (19).

Reply: Thanks for the suggestion. The comma and the point at the end of each equation are checked.

4. The conclusion should be written as the past.

Response: According to the reviewer's suggestion, the conclusion was improved and the conclusion was written in the past tense.

5. The authors can study the following relevant papers and complete their references list: Mathematics and Computers in Simulation 187 (2021) 248-260 and Mathematical Methods in the Applied Sciences 44 (2021) 6247-6258.

Response: The presentation of the paper, especially the introduction and discussion sections, was improved, and the references mentioned were used in addition to other recent papers.

After addressing the above suggestions, I recommend the current manuscript for publication.

Although the manuscript has been well developed, some grammatical errors can be seen in the text. Additionally, there are some editing problems. Therefore, it is suggested to check the whole text in the revised version.

1- Please explain a little about the process of minimizing the answer to equation (53)?

Response:

The equations (46) and (47) are simplified as follows

$\left( a_{1}\frac{d^{4}}{dx^{4}}-a_{2}\frac{d^{2}}{dx^{2}}+a_{3} \right)\bar{w}=-a_{4}\frac{d^{2}\bar{\Psi}}{dx^{2}}-\bar{g}\left( s \right)$	(46)

$-a_{6}\frac{d^{2}\bar{w}}{dx^{2}}=\frac{d^{2}\bar{\Psi}}{dx^{2}}-a_{5}\bar{\Psi}$	(47)

Then the second equation is multiplied by $a_{4}$ and the two equations are added to eliminate the second derivative in $\bar{\Psi}$, we get

$\bar{\Psi}\left( x,s \right)=\frac{1}{a_{4}a_{5}}\left[ -a_{1}\frac{d^{4}\bar{w}}{dx^{4}}+\left( a_{2}+{a_{4}a}_{6} \right)\frac{d^{2}\bar{w}}{dx^{2}}-a_{3}\bar{w}-\bar{g}\left( s \right) \right]$	(53)

2- Based on the variations of the kernel function, which physical phenomenon happens that causes the deflection rapidly rises and reaches to its maximum amount and then gradually falls?

Response:

- The deflection $w$ starts and ends with a value of zero and thus fulfills the boundary conditions ($\left. w\left( x,t \right) \right|_{x=0,L}=0$) of the problem at both ends of the beam ($x=0,L$). That is why the deflection rises quickly, reaches its maximum, and then gradually decreases.
- Also, the presence of thermal loading affecting the first end of the nanobeam makes the deflection maximize near the first side and then gradually decrease.
- As for the peak points in the deflection, it decreases and increases according to the choice of the kernel function.

3- Compared to Figs. 2, 3, and 5, the trend in Fig.4 is different. It is suggested to explain about this fact in the revised version.

Response: The difference in behavior is explained in detail within the manuscript when studying Figure 4. From this explanation we mention:

Figure 4 shows the expansion deformation of the medium adjacent to the first end of the beam as a result of thermal shock from thermal loading, while other regions of the beam suffer from compressive deformation. Deformation is a dynamic process, as it is known. The dilated area steadily expands and grows larger over time.

4- Based on which configuration, it is deduced that rotation has a significant influence on the various physical distributions?

Response: The sentence has been reformulated more clearly. But what is meant is that the angular velocity of rotation has a prominent effect on the distributions of the studied physical fields. For example, the temperature curves increase with the speed of rotation.

5- According to Fig.14, what does happen around 𝑥𝑥 = 0.38, so that the trend of the curves before and after this definite point is different?

Response: The presence of the non-localization factor $\xi$ reduces the amount of the deflection distribution when $x$ is less than 0.33, while its values increase in the other parts of the beam.

In fact, we do not know the physical cause of the phenomenon. However, we believe that the reason for this is the presence of harmonic thermal loading.

6- Conclusion part of the study should be revised. It only reviews the applied process of the study. Meanwhile, it must highlight the main findings and achievements of the proposed study, which have not been presented in this section.

Response: Based on the reviewer's suggestion, the conclusions section was revised. The practical process of the study was reviewed. The main results and achievements of the proposed study, which were not presented in this section, were also highlighted. We now think it will be satisfactory to the reviewer.

7- Since this study is a wave propagation approach, the following papers can be also considered:

| https://doi.org/10.1007/s11071-019-04944-4] | [https://doi.org/10.1016/j.compstruct.2021.114557 |
| --- | --- |

Response: The presentation of the paper, especially the introduction and discussion sections, was improved, and the references mentioned were used in addition to other recent papers.

**Comments from Reviewer 2**

Thank you for taking the time to provide your valuable suggestions and review the manuscript. We have carefully studied your suggestions and made important changes. We have answered your questions and helpful comments in depth. We believe that you will agree with them on the following points:

Reviewer #2: Comments on AOAM-D-21-00825.

The topic of the paper is interesting and worthy of publication. The authors should address the following concerns before final decision.

1- Please explain the advantageous and benefits of the solution method used in this article.

Response: The novelty of the presented work and all the advantages and benefits of the solution method used in this article were explained in the last two paragraphs in the introduction section

2- More up-to-date and relevant articles in the field of size-dependent theories should be added and discussed.

Response: More recent and relevant articles in the field of volume-dependent theories are added and discussed in the Introduction section. Generally the introduction has been updated.

3- Please double-check the grammar in the text.

Response: We thank the reviewer for his notes. The entire manuscript was revised, from the abstract and introduction to conclusions and references. Many parts of it have been reworked.

4- Some parameters in the text need to be described just after their first appearance.

Response: We thank the reviewer for his notes. Parameters were described in the text immediately after they first appeared in the equations.

5- The schematic configuration of the studied problem should be modified to show the real representation of the structure.

Response: The schematic configuration of the studied problem has been modified to show the true representation of the structure as indicated by the reviewer.

The entire manuscript was revised, from the abstract and introduction to conclusions and references. Many parts of it have been reworked.

6- - What kind of material did you use in your simulations?

Response: The physical values of silicon material were used for the purpose of the study and comparison.

**Additional clarifications**

In addition to the above comments, all spelling and grammatical errors pointed out by the reviewers have been corrected.

We look forward to hearing from you in due time regarding our submission and to respond to any further questions and comments you may have.

Sincerely
